# Société D’Anthropologie De Paris

**Published:** 1860-09

**Authors:** 


SOCIÉTÉ D'ANTHROPOLOGIE DE PARIS.—We are pleased to learn that at a sitting of this society, held May 24, 1860, Dr. J. Aitken Meigs, of this city, was elected "Foreign Associate Member," upon the proposition of MM. Geoffroy St. Hilaire, Béclard, and Broca. The only other American, we believe, who has received this honor, is Dr. J. C. Nott, of Mobile.

